# A nutrient mixture reduces the expression of matrix metalloproteinases in an animal model of spinal cord injury by modulating matrix metalloproteinase-2 and matrix metalloproteinase-9 promoter activities

**DOI:** 10.3892/etm.2014.2021

**Published:** 2014-10-15

**Authors:** HONGQI ZHANG, GE CHU, CHAO PAN, JIANZHONG HU, CHAOFENG GUO, JINYANG LIU, YUXIANG WANG, JIANHUANG WU

**Affiliations:** Department of Spine Surgery, Xiangya Spinal Surgery Center, Xiangya Hospital of Central South University, Changsha, Hunan 410008, P.R. China

**Keywords:** nutrient mixture, matrix metalloproteinases, spinal cord injury

## Abstract

This study aimed to determine whether a novel nutrient mixture (NM), composed of lysine, ascorbic acid, proline, green tea extracts and other micronutrients, attenuates impairments induced by spinal cord injury (SCI) and to investigate the related molecular mechanisms. A mouse model of SCI was established. Thirty-two mice were divided into four groups. The sham group received vehicle only. The SCI groups were treated orally with saline (saline group), a low dose (500 μg 3 times/day) of NM (NM-LD group) or a high dose (2,000 μg 3 times/day) of NM (NM-HD group). The levels of mouse hindlimb movement were determined every day in the first week post-surgery. The protein expression levels of matrix metalloproteinase (MMP)-2 and MMP-9 were determined by western blotting. Wild-type and mutant MMP-2- and MMP-9-directed luciferase constructs were generated and their luciferase activities were determined. NM significantly facilitated the recovery of hindlimb movement of the mice in comparison to that in the saline group. The expression levels of MMP-2 in the NM-LD and NM-HD groups were decreased by ~50% compared with the saline group as indicated by western blotting results. The expression levels of MMP-9 in the NM-LD and NM-HD groups were decreased to ~25 and ~10%, respectively. These results suggest that NM significantly inhibits the expression of MMP-2 and MMP-9 proteins. Reverse transcription quantitative polymerase chain reaction results indicated that NM reduced the levels of MMP-2 and MMP-9 mRNA. Furthermore, the luciferase results indicated that site-directed mutagenesis comprising a −1306 C to T (C/T) base change in the MMP-2 promoter and a −1562 C/T base change in the MMP-9 promoter abolished the inhibitory effects of NM on MMP-2 and MMP-9 promoters. These results suggest that NM attenuates SCI-induced impairments in mice movement by negatively affecting the promoter activity of MMP-2 and MMP-9 genes and thus decreasing the expression of MMP-2 and MMP-9 proteins.

## Introduction

Spinal cord injury (SCI), which is often caused by trauma rather than disease, results in various symptoms such as pain, paralysis or movement incontinence (1,2). Treatments of SCI patients include restraining the spine and controlling SCI-induced inflammation to prevent further damage (3,4). Research into treatments for SCI includes the use of controlled hypothermia and stem cells (5,6), although the results of such research have seen little application clinically.

For SCI patients, the secondary injuries include neuronal losses driven by changes in levels of glucose, neuroactive lipids and oxygen, and the release of free radicals, endogenous opioids, amines and amino acids (7–13). SCI-induced changes include activation of several molecular signaling pathways during the first 48 h after SCI. For example, cytoskeletal proteins have various effects on tissue survival, and the expression levels of some genes might be altered, which may have harmful effects on cell survival (14,15). Thus, the identification of novel approaches that target the signaling processes ensuing from traumatic injury to the spinal cord is warranted.

The matrix metalloproteinases (MMPs), especially MMP-2 and MMP-9, play key roles in tumor cell invasion and metastasis by degrading type IV collagen, a major component of the extracellular matrix (16–18). MMP-2 and MMP-9 are secreted as inactive proenzymes and activated by other MMPs or other proteases. MMP-9 is a potent regulator of acute neuroinflammation (19). It was recently reported that reduced MMP-9 expression in the lumbar cord early after thoracic SCI assists the recovery of learning ability in mice (20). Therefore, methods that decrease MMP-9 expression may be useful for treating the impaired wound healing in, for example, diabetic patients. Gene promoter polymorphisms are often important for the roles of proteins. The −1306C genotype ratio of the MMP-2 gene has been found to be significantly higher in patients with lung cancers than in the healthy population, and this genotype is associated with an increased risk of lung cancer (21). The −1562 C to T (C/T) polymorphisms in the MMP-9 gene promoter are considered to be important risk factors associated with primary open-angle glaucoma (22).

It has been reported that a novel nutrient mixture (NM), composed of lysine, ascorbic acid, proline, green tea extracts and other micronutrients, has significant effects on MMP-2 and MMP-9 expression levels both *in vitro* and *in vivo* (23). Therefore, in the present study, a mouse SCI model was established to study the use of NM to treat SCI. NM was administered to the mice and the changes in the expression levels of MMP-2 and MMP-9 were determined.

## Materials and methods

### Animals and surgery

Male CD1 mice (22–28 g), aged 8–10 weeks, were used in this experiment (Vital River Laboratory Animal Technology Co., Ltd, Beijing, China). The mice were kept in cages (5 mice/cage) and maintained in one 12 h light-dark cycle. All animal experiments were conducted according to the ethical guidelines of Xiangya Hospital of Central South University (Changsha, China). Mice were anesthetized with intraperitoneal ketamine and xylazine (20 and 10 mg/kg body weight, respectively). An incision on the midline of the back was made to expose the paravertebral muscles. The spinal cord was exposed by a T5–T8 laminectomy. The SCI was generated by extradural compression of the T6–T7 spinal cord for 1 min with an aneurysm clip. Following surgery, the mice were provided with food and sterile water *ad libitum*.

### Experimental grouping and the nutrient mixture (NM) treatments

NM was prepared according to previously reported methods (23). NM was composed of the following ingredients: 700 mg vitamin C, 1,000 mg L-lysine, 750 mg L-proline, 500 mg L-arginine, 200 mg *N*-acetyl cysteine, 1,000 mg standardized green tea extract, 30 μg selenium, 2 mg copper, and 1 mg manganese.

A total of 32 mice were grouped into four groups (8 mice/group) for this experiment, which comprised one sham and three experimental groups. The mice in the sham group were subjected to laminectomy only, without SCI being generated. The other 24 mice were allocated into the three experimental groups treated with different dosages of NM or vehicle (saline). The SCI model mice received oral NM or saline in the 3 days following SCI. The sham group received vehicle only. The SCI groups were treated orally with saline, a low dose (500 μg 3 times/day) of NM (NM-LD) or a high dose (2,000 μg 3 times/day) of NM (NM-HD).

### Movement function evaluation

The Basso mouse scale (BMS) for locomotion was used to evaluate the level of motor dysfunction following SCI (24). Prior to injury, the mice were examined to ensure that they were all at normal level with a score of 21. In 7 days after the completion of NM, the mice in every group were scored. The scoring was initiated 3 days following SCI and was conducted for 7 days. Scores for each hindlimb were averaged for each day.

### Western blotting

Mice were euthanized after completion of the experiments and the spinal cords were quickly dissected, frozen and stored at −80°C. Segments from L4–L5 were homogenized in lysis buffer with the addition of protease inhibitor mixture (Roche Diagnostics, Basel, Switzerland). Total proteins were separated on 10% SDS/PAGE gels, and then analyzed by immunoblotting. The primary antibodies against MMP-2, MMP-9 and β-actin were purchased from Santa Cruz, USA (anti-MMP-2, cat. no. sc-53630, 1:200; anti-MMP-9, cat. no. sc-21733, 1:200; anti-β-actin, cat. no. sc-130301, 1:10,000). Secondary antibodies used in this study were goat anti-mouse horseradish peroxidase-conjugated immunoglobulin G (IgG-HRP; cat. no. sc-2005, 1:10,000; Santa Cruz Biotechnology, Inc., Dallas, TX, USA). The bound antibodies were detected using an electrochemiluminescence (ECL) system (Pierce Biotechnology Inc., Rockford, IL, USA). Image quantifications were performed using ImageQuant software (GE Healthcare, Uppsala, Sweden). The experiments were repeated at least three times.

### Reverse transcription quantitative polymerase chain reaction (RT-qPCR)

Mice were euthanized by CO_2_ inhalation following completion of the experiments and the spinal cords were quickly dissected for RNA isolation using the RNeasy kit (Qiagen, Valencia, CA, USA) according to the manufacturer’s instructions. One microliter of RNA was reverse transcribed into cDNA using random primers with a Reverse Transcription II system (Promega Corporation, Madison, WI, USA), according to the manufacturer’s instructions. PCR was conducted using an ABI Prism Sequence Detection System (Applied Biosystems, Foster City, CA, USA). A VIC^®^-labeled probe (cat. no. 4310884E; Applied Biosystems) was used to quantify the expression of endogenous GAPDH mRNA, which was used as an internal control. Amplification of the MMP-2 and MMP-9 cDNAs and the endogenous GAPDH cDNA were determined using FAM™ and VIC fluorescence, respectively. The relative amounts of MMP-2 and MMP-9 transcripts were expressed as ratios relative to the levels of GAPDH mRNA. The experiments were repeated independently at least three times. The primers used for MMP-2 were 5′-GGAGCA CGTCATGCAC and 5′-AGACACGCTAGTAGGC, and for MMP-9 were 5′-CACCACTGCAATTGCG and 5′-CACCAT CTCATACGT GAG.

### Construction of firefly luciferase constructs driven by human MMP-2 or MMP-9 promoters

A 1.6 kb segment at the 5′-flanking region of the human MMP-2 gene or a 1.7 kb segment at the 5′-flanking region of the human MMP-9 gene was generated by PCR using primers from the human MMP-2 gene (Gene ID: 4313) and MMP-9 gene (GenBank accession no. D10051). The primers used were: MMP-2 forward, 5′-AGCTAAGGCTTAGGGTACGGC; MMP-2 reverse, 5′-GCGTTAACGGACGCTAGCTAG; MMP-9 forward, 5′-TGCACCGTGCATACCTTAG; and MMP-9 reverse, 5′-AGGGGCTGCCAGAAGCTTATGGT. The pGL2-Basic vector (Promega Corporation) containing a polyadenylation signal upstream from the luciferase gene was used to construct expression vectors by subcloning PCR-amplified DNA of MMP-2 or MMP-9 promoters into the *Sac*I/*Hin*dIII site of the pGL2-Basic vector. Point mutations at the loci of −1306 C/T and −1562 C/T were made using the Site-Directed Mutagenesis kit (Agilent, Santa Clara, CA, USA). Clones were confirmed by DNA sequencing.

### Transfections and luciferase gene assays

In brief, HeLa cells were plated onto six-well plates at a density of 2×10^5^ cells/well and grown overnight. Cells were cotransfected with 1 μg construct template (either wild-type or mutant pMMP-2-LUC and pMMP-9-LUC constructs) and 1 μg pCMV-β-galactosidase construct using Lipofectamine reagent (Life Technologies, Grand Island, NY, USA). After 4 h, cells were treated with vehicle only (sham group), saline (saline group), 100 μg/ml NM (NM-LD group) or 500 μg/ml NM (NM-HD group). Luciferase and β-galactosidase activity was determined according to the manufacturer’s instructions and the luciferase activity of each sample was normalized to β-galactosidase activity to calculate the relative luciferase activities. Data are the mean ± SD from at least five experiments. Luciferase assay and β-galactosidase assay systems were purchased from Promega Corporation.

### Statistical analysis

The experimental data are expressed as mean ± standard deviation. Statistical software (SPSS version 10.0; SPSS, Inc., Chicago, IL, USA) was used for independent sample t-tests, followed by one-way variance analysis. In all analyses, P<0.05 was considered to indicate a statistically significant difference.

## Results

### SCI-related activity was attenuated by treatment with NM

**Figure 1:**
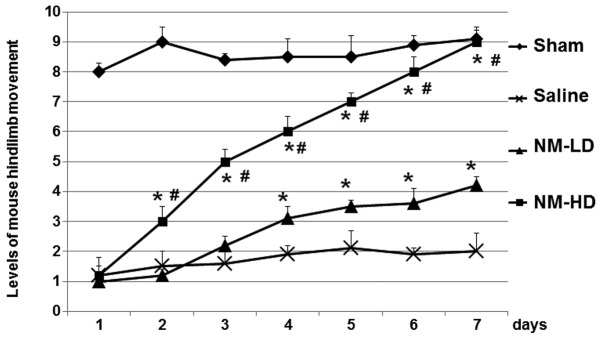
NM attenuates the impairments in levels of mouse hindlimb movement generated by SCI. Thirty-two mice were divided into four groups (8 mice/group). The sham group received vehicle only. The SCI groups were treated orally with saline (saline group), a low dose (500 μg 3 times/day) of NM (NM-LD group) or a high dose (2,000 μg 3 times/day) of NM (NM-HD group). The levels of mouse hindlimb movement were determined every day in the first week post-surgery. The BMS scores were ranged from 1 (complete paralysis) to 9 (normal movement of the hindlimbs). Scores were assigned for the two hindlimbs of each mouse, and the two scores were averaged to obtain a mean value for each mouse. Data are mean values of 8 mice in each group. NM, nutrient mixture; SCI, spinal cord injury; LD, low dose; HD, high dose; BMS, Basso mouse scale. ^*^P<0.05 vs. saline; ^#^P<0.05 vs. NM-LD.

Changes in hindlimb movement of the mice, as an indicator of the SCI-related consequences, were determined. As shown in Fig. 1, the hindlimb movements of mice in the sham, NM-LD and NM-HD groups were significantly decreased at day 1 after SCI, suggesting that the model was successfully established. The function levels of mice in the saline group were not recovered within 7 days following the surgery. However, the hindlimb movement levels of mice in the NM-LD and NM-HD groups were significantly recovered when compared with those in the saline group (Fig. 1). Furthermore, the recovery was better in the NM-HD group than in the NM-LD group. These results suggest that NM significantly increased the recovery of hindlimb movement of the mice in comparison with that in the saline group.

### NM decreases the expression of MMP-2 and MMP-9 proteins

**Figure 2:**
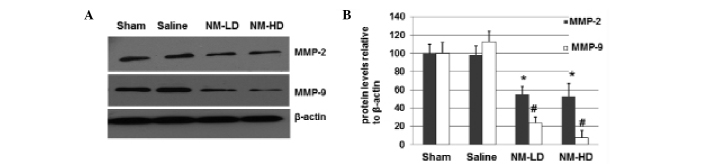
NM decreases the expression of MMP-2 and MMP-9 proteins. Total proteins were harvested and separated on 10% SDS/PAGE gels, and then analyzed by immunoblotting. (A) Representative immunoblots. Detection of β-actin was used as a loading control. (B) The experiments were repeated independently at least three times. Image quantifications were performed using ImageQuant software. Data are mean values of 8 mice in each group. NM, nutrient mixture; MMP, matrix metalloproteinase; LD, low dose; HD, high dose. ^*^P<0.05 vs. the saline group; ^#^P<0.05 vs. the saline group.

To determine whether NM is able to decrease the expression of MMP-2 and MMP-9, mice in the sham, saline, NM-LD and NM-HD groups were euthanized and the spinal cords were quickly dissected for immunoblotting analyses. As shown in Fig. 2, the expression levels of MMP-2 in the NM-LD and NM-HD groups were decreased by ~50% compared with the saline group. The expression levels of MMP-9 in the NM-LD and NM-HD groups were decreased to ~25 and ~10%, respectively. These results suggest that NM significantly inhibits the expression of MMP-2 and MMP-9, with greater inhibitory effects on MMP-9 expression than on MMP-2 expression.

### NM decreases the levels of MMP-2 and MMP-9 mRNA

**Figure 3:**
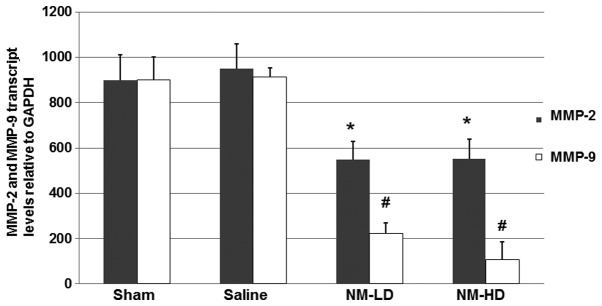
Detection of MMP-2 and MMP-9 mRNA by quantitative reverse transcription polymerase chain reaction (RT-qPCR). Total RNAs were harvested from tissues. RT-qPCR was performed to analyze the MMP-2 and MMP-9 mRNA levels in the spinal cords. The levels (mean value) of MMP-2 and MMP-9 transcripts were calculated. Error bars show the standard deviation. The experiments were repeated at least three times. NM, nutrient mixture; MMP, matrix metalloproteinase; LD, low dose; HD, high dose. ^*^P<0.05 vs. the saline group; ^#^P<0.05 vs. the saline group.

To further study the mechanism underlying the inhibitory effects of NM on the increased MMP-9 expression, total RNAs were harvested from the dissected spinal cords of the mice and RT-qPCR was performed to analyze the mRNA levels of MMP-2 and MMP-9. As shown in Fig. 3, the RT-qPCR results indicated that NM significantly inhibited the expression of MMP-2 and MMP-9 mRNA, respectively. These results suggest that NM inhibits the expression of MMP-2 and MMP-9 via a transcriptional mechanism.

### NM decreases the transcriptional promoter activities of MMP-2 and MMP-9 mRNA

**Figure 4:**
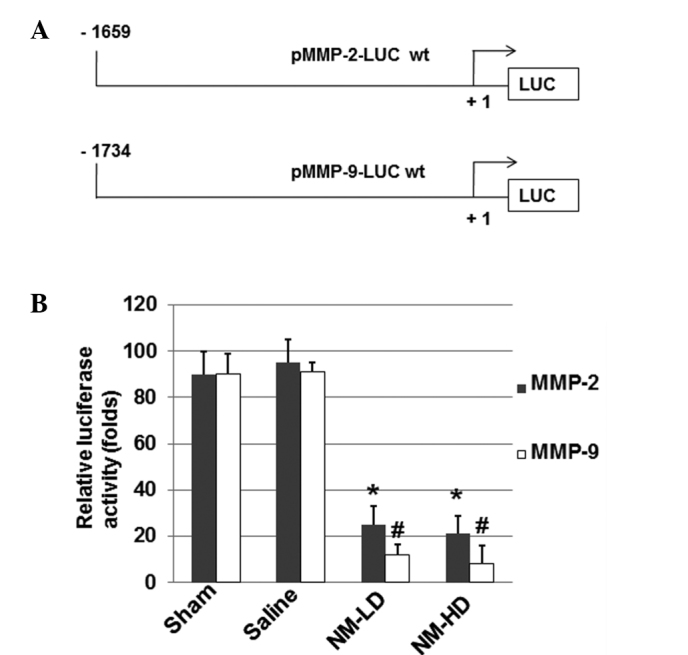
Effect of NM on human MMP-2 and MMP-9 promoter activities. (A) Wild-type MMP-2 and MMP-9 promoter-directed luciferase constructs were constructed. (B) HeLa cells were co-transfected with 1 μg wild-type pMMP-2-or wild-type pMMP-9-LUC construct in addition to 1 μg pCMV-β-galactosidase construct. After 4 h, cells were treated with vehicle only (sham group), saline (saline group), 100 μg/ml NM (NM-LD group) or 500 μg/ml NM (NM-HD group). Luciferase and β-galactosidase activity was determined and the luciferase activity of each sample was normalized to β-galactosidase activity to calculate the relative luciferase activities. Data are the mean ± SD from at least five experiments. NM, nutrient mixture; MMP, matrix metalloproteinase; LD, low dose; HD, high dose; LUC, firefly luciferase gene.^*^P<0.05 vs. the saline group; ^#^P<0.05 vs. the saline group.

To further determine whether NM affects the transcriptional activity of MMP-2 and MMP-9 mRNAs, luciferase constructs driven by MMP-2 or MMP-9 promoter sequences were prepared (Fig. 4A) and investigated using a luciferase assay. As shown in Fig. 4B, NM significantly inhibited the MMP-2 and MMP-9 promoter-directed luciferase activities, respectively (P<0.05) when compared with those in the sham and saline groups. These results suggest that NM inhibits the expression of MMP-2 and MMP-9 via a mechanism related to the regulation of their gene promoters.

### Site-directed mutagenesis abolishes the inhibitory effects of NM on MMP-2 and MMP-9 promoters

**Figure 5:**
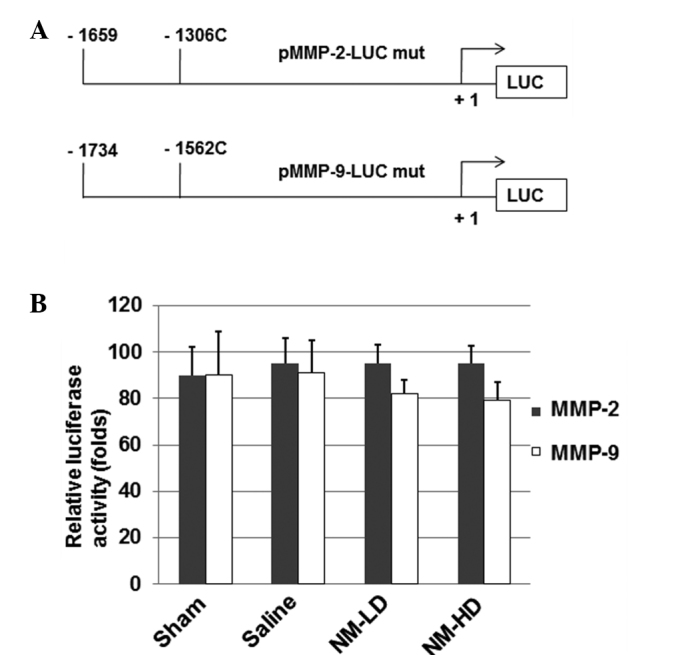
Site-directed mutations on human MMP-2 and MMP-9 promoters abolish the effect of NM. (A) The −1306 C/T and −1562 C/T mutations were generated on the luciferase constructs. (B) HeLa cells were co-transfected with 1 μg mutant pMMP-2-LUC or mutant pMMP-9-LUC construct in addition to 1 μg pCMV-β-galactosidase construct. After 4 h, cells were treated with vehicle only (sham group), saline (saline group), 100 μg/ml NM (NM-LD group) or 500 μg/ml NM (NM-HD group). Luciferase and β-galactosidase activity was determined and the luciferase activity of each sample was normalized to β-galactosidase activity. Data are the mean ± standard deviation from at least five experiments. NM, nutrient mixture; MMP, matrix metalloproteinase; LD, low dose; HD, high dose; C/T, C to T; LUC, firefly luciferase gene.

Site-directed mutagenesis was performed to generate the −1306 C/T base change on the MMP-2 promoter and the −1562 C/T base change on the MMP-9 promoter in the luciferase constructs (Fig. 5A). The luciferase assay results (Fig. 5B) indicate that NM did not significantly inhibit the MMP-2 and MMP-9 promoter-directed luciferase activities when compared with those in the sham and saline groups. These results suggest that these loci are important for the inhibitory effect of NM on MMP-2 and MMP-9 gene expression.

## Discussion

The reduction of further damage is very important in the treatment of patients with SCI. In the present study, a mouse model of SCI was established to study the use of NM in the treatment of SCI. The mice were treated with NM and the changes in the expression levels of MMP-2 and MMP-9 were detected. It was found that NM significantly attenuated the SCI-induced impairment in mice movement and also decreased MMP-2 and MMP-9 expression in a dose-dependent manner.

MMP-2 and MMP-9 are secreted as inactive proenzymes and activated by other MMPs or other proteases. As a potent regulator of acute neuroinflammation (19), MMP-9 has recently been found to be able to reduce MMP-9 expression in the lumbar cord early after thoracic SCI, suggesting that MMP-9 might be helpful for the recovery of learning ability in mice (20). The finding in the present study that NM decreases MMP-9 expression in a dose-dependent manner improves the understanding of the roles of MMP-9. It was noted that the inhibitory effect of NM on MMP-9 protein expression was more evident than that on MMP-2. The reason underlying this difference remains to be studied in the future.

In SCI patients, secondary injuries are often induced. The SCI-induced secondary injuries have various symptoms, including the neuronal losses driven by changes in the levels of glucose, neuroactive lipids and oxygen, and the release of free radicals, endogenous opioids, amines and amino acids (7–13). In the present study, the recovery of hindlimb movement of the mice treated with NM in comparison with that in the saline group was used as an indicator of the effect of NM on SCI. The BMS for locomotion was used to evaluate the level of motor dysfunction following SCI. Scores for each hindlimb were averaged for each day. It was found that in 7 days, a high dose (2,000 μg 3 times/day) of NM significantly facilitated the recovery of mouse hindlimb movement generated by SCI, although a low dose (500 μg 3 times/day) of NM also had detectable effects from the fourth day after SCI. These findings imply that NM may have an important role in the clinic upon further studies in the future.

Changes in protein expression are often associated with the promoter activities of genes. In the present study, luciferase experiments were performed to investigate the effects of NM on MMP-2 and MMP-9 promoter activities. The results suggest that the mutations on the −1306 C locus of the MMP-2 promoter and the −1562 C locus of the MMP-9 promoter abolished the inhibitory effects of NM on MMP-2 and MMP-9 promoters. Since numerous cellular protein factors, such as AP-1 and CREB (25,26), can bind to these sites on the promoters, further studies to identify the cis-acting elements and trans-acting factors that may be involved in the regulation of MMP-2 and MMP-9 expression are planned.
